# Lemierre's Syndrome Complicating Pregnancy

**DOI:** 10.1155/2007/68084

**Published:** 2007-06-28

**Authors:** M. Thompson, A. O. Awonuga, J. Bell, C. Ray, M. T. Awonuga, A. Helfgott

**Affiliations:** ^1^Division of Maternal Fetal Medicine, Department of Obstetrics and Gynecology, Medical College of Georgia, 1120 15th Street, Suite BB7513, Augusta, GA 30912, USA; ^2^Division of Neonatology, Department of Pediatrics, Medical College of Georgia, 1120 15th Street, BIW 6033, Augusta, GA 30912, USA

## Abstract

Lemierre's syndrome is an anaerobic suppurative thrombophlebitis involving the internal jugular vein secondary to oropharyngeal infection. There is only one previous case report in pregnancy which was complicated by premature delivery of an infant that suffered significant neurological damage. We present an atypical case diagnosed in the second trimester with a live birth at term. By reporting this case, we hope to increase the awareness of obstetricians to the possibility of Lemierre's syndrome when patients present with signs of unabating oropharyngeal infection and pulmonary symptoms.

## 1. INTRODUCTION

Lemierre's syndrome (LS) is an anaerobic suppurative thrombophlebitis involving the internal jugular vein, most often as a complication of pharyngeal and dental infection. Septicemia documented by at least one positive blood culture and one or more metastatic infective focus completes the features of this syndrome. The disease most commonly affects teenagers and young adults; therefore, it can occur in association with pregnancy. However, a PubMed, Ovid and Embase search from 1950 to March 2007, using search terms “Lemierre's syndrome and Lemierre's disease, pregnancy, gestation and cyesis,” revealed only one case in which the pregnancy was complicated by premature delivery of an infant that suffered significant neurological deficits at 26 weeks [1]. We present an atypical case diagnosed by chance in a previously healthy, young woman who developed the syndrome in the second trimester of pregnancy.

## 2. CASE REPORT

A 21-year-old white female, G5P4004, at 22 weeks was transferred to our hospital for evaluation of worsening sepsis. She had no significant obstetrical, medical, or drug history. She had presented to her local health facility with a three-day history of headache, nonproductive cough and fever. She was evaluated and discharged home twice in 3 days with these symptoms. For the next week, her symptoms persisted and worsened; therefore, she was admitted to another medical facility in septic shock. She was given intravenous fluids, placed on broad-spectrum antibiotics and required a dopamine drip due to persistent hypotension. She demonstrated pancytopenia (Hb, 7 g/dL; WBC, 1.7 × 10^9^/L; Platelets 53 × 10^9^/L) and received 2 units of packed red blood cell transfusion prior to transfer to our facility.

On presentation, she was somnolent but arousable. She was pale but not jaundiced and had mild expiratory wheezes. Her temperature was 36.2°C, pulse was 88 beats/min, blood pressure was 96/54 mmHg, respiratory rate was 30/min with O_2_ saturation of 100% on 40% of oxygen. Her pupils were equal and reactive to light. She had no lymphadenopathy. Both heart sounds were heard with no murmurs. Her chest was symmetrical with good air entry and bilateral scattered rhonchi were heard. Her abdomen was soft and nontender. Her fundal height was compatible with her dates and fetal heart tone was 155 beats/min. Her cranial nerves II through XII were intact.

The patient was admitted to the Medical Intensive Care Unit with a diagnosis of septic shock. Intravenous cefepime, azithromycin, and levofloxacin were started after cultures were sent. On hospital day two, she developed respiratory distress requiring intubation and mechanical ventilation. During an attempt at placement of a left internal jugular intravenous access, copious amounts of purulent material exuded. CT revealed suppurative mastoiditis with erosion of the mastoid bone towards the left transverse sigmoid sinus; secondary septic thrombophlebitis involving the left transverse sinus, sigmoid sinus, internal jugular and left brachiocephalic veins. In addition, bilateral pleural effusions and multiple bilateral patchy areas of focal airspace disease were noted, implying a septic phenomenon to the lungs. A diagnosis of LS was made. Otolaryngology and Neurosurgery were consulted and patient underwent left mastoidectomy, evacuation of copious abscess deep in the left mastoid antrum, right myringotomy, and tube placement. Cultures grew *Peptostreptococcus anaerobius, Bacteroides fragilis*, and *Eikenella corrodens*. Infectious disease service advised that her antibiotics be changed to piperacillin/tazobactam and metronidazole. Although her sepsis improved, it was difficult to wean her off the ventilator due to hemodynamic instability and atelectasis from right-sided pneumothorax. A chest tube was placed and on removal, residual right lower lobe atelectasis persisted which resolved after a bronchoscopic examination to clear out secretions. On extubation, the patient made a gradual but steady recovery and was discharged home after a 23-day hospital stay. Arrangements were made for her to continue with physical therapy and intravenous antibiotics for 6 weeks.

Throughout her entire hospital course, fetal heart tones were obtained every shift. Ultrasounds examination confirmed her dates and normal anatomy. Subsequent antepartum fetal surveillance was with serial nonstress tests and biophysical profiles. Follow-up growth scans suggested intrauterine growth restriction, necessitating induction of labor at 37 weeks. The patient had a successful induction with spontaneous vaginal delivery of a live female infant weighing 2892 grams with Apgar scores of 9 at one minute and 9 at five minutes.

## 3. DISCUSSION

Lemierre's Syndrome is a potentially fatal multisystemic disease resulting from complication of anaerobic infection of the head and neck usually in previously healthy individuals. Although few cases had been described prior, it was Lemierre [2], a professor of bacteriology and infectious disease at the Claude Bernard Institute in Paris, who first wrote a comprehensive review of this disease in 1936 and subsequently earned the name by which the syndrome is now known. The infectious causes are diverse and are part of the normal flora of the mouth, genital, gastrointestinal, and upper respiratory tracts. They often include anaerobic, but may also include aerobic organisms. *Fusobacterium necrophorum*, a highly virulent obligatory anaerobe, is often but not invariably associated with this condition and was not isolated in our case. The original infection is usually a tonsillitis, pharyngitis, or odontogenic infection but has also been described following penetrating oropharyngeal trauma [3]. Our patient did not volunteer the typical history of initial oropharyngeal infection, neither was her neck tender despite the presence of mastoid abscess. This is not surprising because unlike infection and thrombophlebitis involving the anterior compartment of the lateral pharyngeal space, that involving the posterior space may remain asymptomatic until the disease progresses systematically to include septic pulmonary embolism [4]. Had the placement of an internal jugular venous catheter not been performed in our case, the diagnosis might have been delayed with serious consequences. However, this led to the chain of events that revealed the diagnosis and resulted in a successful outcome by enabling the removal of the primary focus of infection. The advent of high-resolution computed tomography for delineating the extent of thrombophlebitis has led to more cases being diagnosed and should be performed as early as possible. Septic embolization occurs most commonly to the lungs but may involve virtually any organ.

The disease most commonly affects teenagers and young adults; therefore, it is not difficult to envisage its occurrence in association with pregnancy. McLean and Tyler [1] reported a nearly fatal case in a 23-year-old primigravida that resulted in premature delivery of an infant that suffered significant neurological deficits at 26 weeks. In that case, LS was complicated by cardiopulmonary collapse due to mediastinitis, pericardial effusion and tamponade that required mechanical ventilation, cardioversion, and emergent pericardiocentesis. Fusobacteria are known to play a role in the parthogenesis of preterm labor. The traditional view is that microorganisms gain access to the amniotic cavity after rupture of membranes; however invasion of the amniotic cavity can take place prior to this. Romero et al. [5] found that 13.6% of women with preterm labor and intact membranes had a positive amniotic fluid culture, and that such women are more likely to rupture than those with a negative culture. However, our case fortunately resulted in a live birth of an apparently healthy child at term. The natural history of cases described in the literature is similar to that encountered in these two pregnant cases (this case and the one before it), hence pregnancy cannot be said to aggravate the course of the disease. Aside from sepsis, the associated pleuropulmonary disease common with this syndrome has implication for the pregnant patient. Decrease in colloid oncotic pressure encountered in pregnancy, in conjunction with the added effect of acute lung injury causing lung exudation, surfactant loss and diminished lung volume, and vascular shunting, can result in significant hypoxemia. Intubation and mechanical ventilation is preferred in pregnant women if respiratory failure exists or is imminent to avoid defective oxygen delivery to the baby [6].

Management necessitates a multidisciplinary approach. An extended course of intravenous antibiotics based on the results of cultures is often required and although it is currently advocated that it be continued for 6 weeks, this is based solely on expert opinion [7]. Goldenberg et al. [4], in their study of the disease in the pediatric age group, found thrombophilic abnormalities in all investigated cases of LS in their series but in only one patient did the abnormality persisted after they were retested 2 to 6 months after diagnosis. These authors suggested that these thrombophilic abnormalities might be an epiphenomenon of the acute inflammatory prothrombotic state rather than an intrinsic hypercoagulability in these patients. Therefore, routine tests for thrombophilia may not be justified or cost effective until more data are available. The role of anticoagulant therapy in Lemierre's syndrome is controversial butmay be beneficial [8], however, no control trial exists studying the efficacy of anticoagulant therapy in this condition. Because of the possibility of extension of the infective agents, some suggest that anticoagulant therapy should be reserved for thrombosis retrograde to the cavernous sinus [9].

The likely effect of pregnancy on the course of disease and vice-versa cannot be ascertained from this single case and the one before it. Nevertheless, the severe and sometimes life-threatening nature of this syndrome necessitates an enhanced awareness by primary care and emergency room physicians and obstetricians. When a previously healthy patient presents with sepsis following an unabating oropharyngeal infection in association with pulmonary symptoms, a chest X-ray should be performed at the minimum followed by a CT scan of the head, neck, and chest if LS cannot be excluded. Management as outlined by our case and those of others necessitates a multidisciplinary approach if mortality is to be reduced.
